# Sohlh1 and Lhx8 are prominent biomarkers to estimate the primordial follicle pool in mice

**DOI:** 10.1186/s12958-023-01097-3

**Published:** 2023-05-16

**Authors:** Li Liu, Biting Liu, Lian Wang, Caixia Li, Yang Zhou, Jihui Zhu, Jinye Ding, Shupeng Liu, Zhongping Cheng

**Affiliations:** 1 Department of Obstetrics and Gynecology, Shanghai Tenth People’s Hospital, School of Medicine, Tongji University, Shanghai, 200072 China; 2 Institute of Gynecological Minimally Invasive Medicine, School of Medicine, Tongji University, Shanghai, 200072 China

**Keywords:** *Sohlh1*, *Lhx8*, Primordial follicle pool, Biomarkers, Clinical assessment

## Abstract

Efficient evaluation of the primordial follicle pool (PFP) of mammalian models is an essential subject in biomedical research relating to ovarian physiology and pathogenesis. Our recent study has identified a gene signature including *Sohlh1*, *Nobox*, *Lhx8*, *Tbpl2*, *Stk31*, *Padi6*, and *Vrtn* strongly correlated with ovarian reserve by using bioinformatics analysis. Aimed to investigate the validity of these candidate biomarkers for evaluating the PFP, we utilized an OR comparison model to decode the relationship between the numbers of PFP and candidate biomarkers in the present study. Our results suggest that these biomarkers *Sohlh1, Nobox, Lhx8, Tbpl2, Stk31, Padi6*, and *Vrtn* possess independent potential to evaluate the number of the PFP. And the combination of *Sohlh1* and *Lhx8* can be used as the optimal biomarkers for rapid assessment of the PFP in the murine ovary. Our findings provide a new perspective for evaluating the PFP of the ovary in animal studies and the clinic.

**Supplementary Information:**

The online version contains supplementary material available at 10.1186/s12958-023-01097-3.

## Introduction

It is well-known that the primordial follicle pool in mammals represents the ovarian reserve (OR) that is fixed after birth and not renewed [1]. As time goes on, the primordial follicle pool (PFP) gradually decreases with continued atresia and recruitment until depleting after menopause [2]. The depletion of the PFP accelerates when the ovaries are exposed to chemotherapy, environmental chemicals, and numerous pathological factors, leading to primary ovarian insufficiency (POI)[3].

As the basic functional unit of the ovary, the primordial follicle is a critical subject for studies related to ovarian physiology and pathogenesis [4–6]. The measurement of PFP is accomplished based on microscopic recognition and individual counting by the visual observation of researchers on serial tissue Sects. [7–9]. The workload for follicle counting is tremendous in animal studies with large sample requirements. Critically, this method is quite subjective, and the number of PFP reported by different teams varies dramatically. It is considered that this variation in the follicular number reported reflects inherent biases of this counting technique [7]. Furthermore, with the application of new assisted reproductive technologies (ART) such as ovarian tissue cryopreservation (OTC), there is a challenge to assess PFP level efficiently and accurately in ovarian tissue slices in clinical practice [10–12]. Therefore, an objective, precise, and simplified approach to assessing PFP at the histological level is urgently needed.

High throughput sequencing has advanced dramatically in recent years, providing new approaches for identifying biomarkers [13]. Our recently published study has found a gene signature strongly correlated with OR based on the transcriptomic data of bulk and single-cell RNA-seq. Genes including *Sohlh1, Nobox, Lhx8, Tbpl2, Stk31, Padi6*, and *Vrtn* could be potential biomarkers to evaluate the PFP in humans and mice ovarian tissues [14].

In the present study, we aimed to investigate the validity of these seven candidate biomarkers and identify the optimal combination of biomarkers for evaluating the PFP, hoping to provide a novel and complementary method for the rapid assessment of the PFP.

## Materials and methods

### Animals and sample preparation

Different aged C57BL/6L female mice with specific pathogen-free conditions were purchased from Vital River Laboratory Animal Technology Corp. The mice were raised in an environment with a temperature of between 18 and 23 °C and humidity of between 40 and 60% under 12-hour light/dark cycles. According to the previous study, bilateral ovariectomy was performed under the microscope according to the previous study [15]. Both ovaries of each mouse were dissected intact, and one side was randomly selected for serial histological analysis, while the entire opposite ovary was subjected to RNA extraction. Three mice were excluded due to failure of sample collecting or RNA extraction. Animal experiments were approved by the Animal Ethics Committee of Shanghai Tenth People’s Hospital (No. SHDSYY-2021-Y0688).

### Quantitative real-time PCR

The total RNA extraction was completed with RNAiso Plus (TaKaRa, Shiga, Japan). RNA quality and quantity were measured by NanoDrop2000 (Thermo Scientific, Wilmington, DE, USA). The RNA was reverse-transcribed into complementary DNA using PrimeScript RTMaster Mix (TaKaRa, Japan) according to the manufacturer’s instructions. Quantitative real-time PCRs (qRT-PCR) were performed on QuantStudio Dx (ABI, America) with SYBR Premix ExTaq kit (Takara, Shiga, Japan). The thermal cycler conditions were as follows: 30 s at 95.0 °C for cDNA denatured, followed by 40 cycles of 15 s at 95 °C and 60 °C for the 34 s. Verification of specific product amplification was performed by dissociation curve analysis. The mRNA relative expression was calculated by the 2^^−ΔCt^ method with Gapdh used as an internal control [16, 17].Primer sequences for each gene were listed in Supplementary Table 1.

**Table 1:** ROC analysis of the expression of candidate genes for assessing OR levels

Gene	AUC	S.E.	*Cut-off*	*Sensitivity (%)*	*Specificity (%)*	*P*
*Nobox*	0.915	0.060	0.00185771	96.3	80	**< 0.001**
*Stk31*	0.857	0.072	0.0002692	77.8	80	**< 0.001**
*Sohlh1*	0.993	0.010	0.00015527	92.6	100	**< 0.001**
*Vrtn*	0.778	0.088	0.00034877	100	50	**0.010**
*Padi6*	0.811	0.083	0.0064735	96.3	60	**0.004**
*Tbpl2*	0.759	0.085	0.00028304	59.3	90	**0.017**
*Lhx8*	0.837	0.078	0.0009555	85.2	80	**0.002**

AUC, area under curve; S.E, standard error; P values < 0.05 are in bold

### Histological analysis and follicle counting

Ovaries were fixed in 4% buffered paraformaldehyde for one day, followed by paraffin embedding. Each murine ovary was serially sectioned at 5-µm thickness and mounted on glass slides. The slides were stained with hematoxylin and eosin (Merck, Darmstadt, Germany) and analyzed for morphological grading and follicle density count. To avoid miscounting, only follicles surrounded by a single flattened granulosa layer were also scored as primordial follicles [18], and only grade1 primordial follicles were counted in [19].

### Statistical analysis

Data analysis was performed using SPSS statistical software (IBM, New York, NY, United States). Quantitative data were expressed as the standard deviation of the mean. Data were tested for normal distribution using the Kolmogorov-Smirnov test. The data analysis between the two groups was assessed with Student’s t-test or Mann–Whitney U test according to the normality of the data distribution. Receiver Operating Characteristic (ROC) curve was calculated in SPSS with Mann-Whitney U (Wilcoxon) test. The cut-off point was calculated with the Youden index based on the ROC analysis. Bivariate correlations were analyzed with the Pearson correlation test. Univariate and multivariate stepwise linear regression analyses performed with the independent variables reference correlation analysis results, including all candidate genes. The variance inflation factor (VIF) was used to measure the collinearity of variables, and variables with a VIF smaller than 5 were included in the analysis. P values < 0.05 were considered statistically significant.

## Result

### Establishment of ovarian reserve comparison murine model

**Fig. 1:**
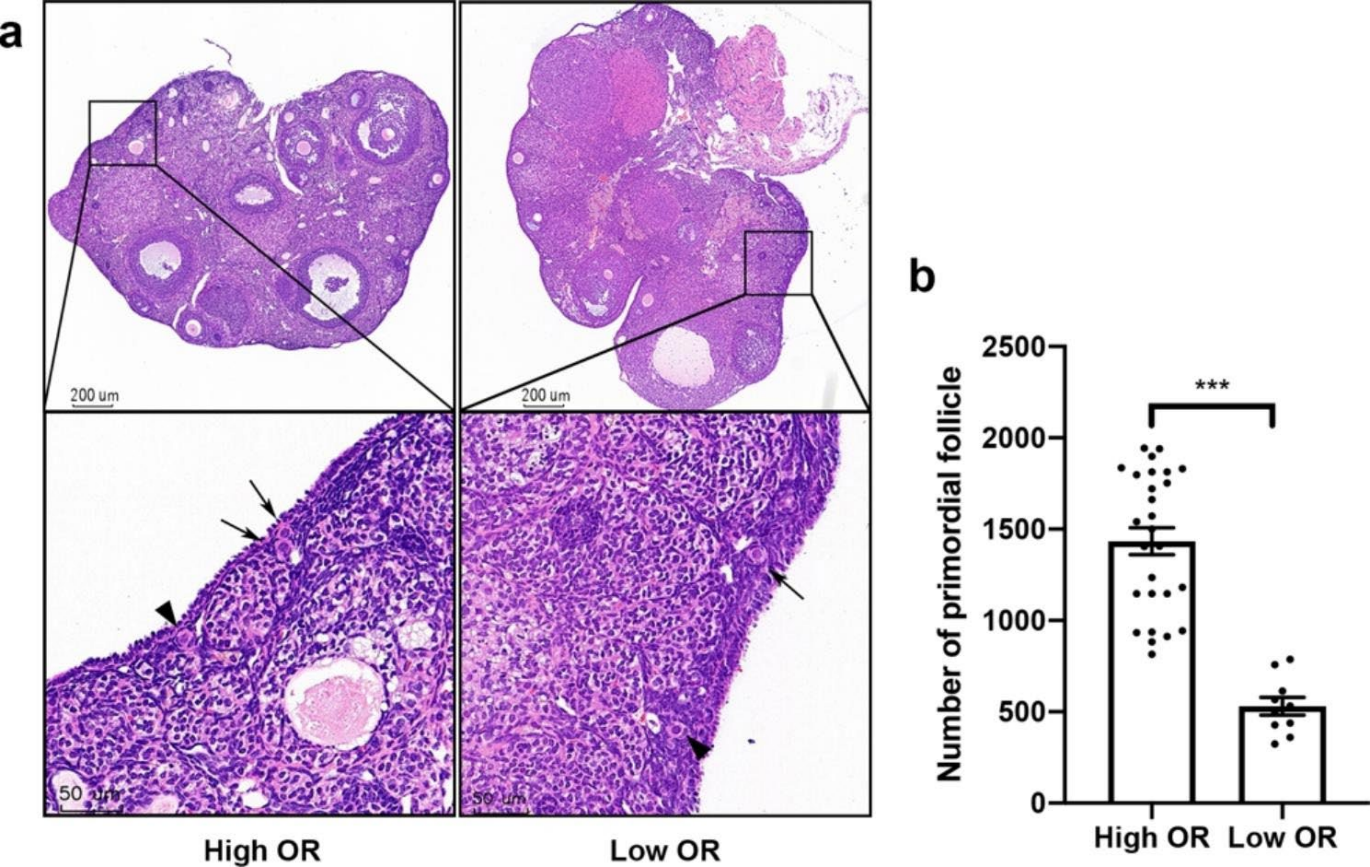
Histological counting of primordial follicles in **OR** comparison murine model. **(a)** Representative histological images of the ovaries from different groups. Arrows indicate primordial follicles that morphologically conform to the G1 stage. Arrowheads indicate that the flattened pre-granulosa cells of Transitional follicles become cubic. **(b)** The numbers of primordial follicles by Histological counting in murine. *** P < 0.001

It is well known that ovarian reserve is progressively depleted with age in mammals. Over the past decades, rodents have been widely used in ovarian-related biological research as an essential component of mammalian models. In this study, we attempt to establish a comparative model of OR depending on the age of the mice [20]. A total of 37 mice were divided into the high OR group (week-old 12.93 ± 1.66) and the low OR group (week-old 36.00 ± 0.67) based on previous estimates of mouse reproductive age. As shown in Fig. 1a, primordial follicle counts were completed by histology and morphological analysis, and only grade1 primordial follicles were counted in [19]. The number of primordial follicles in the high OR group was significantly more than that in the low OR group (1433.95 ± 73.47 vs. 530.54 ± 49.24; *P* < 0.001) (Fig. 1b), which indicates the effective establishment of the animal model for OR comparison.

### Validation of potential biomarkers to evaluate OR capability

**Fig. 2:**
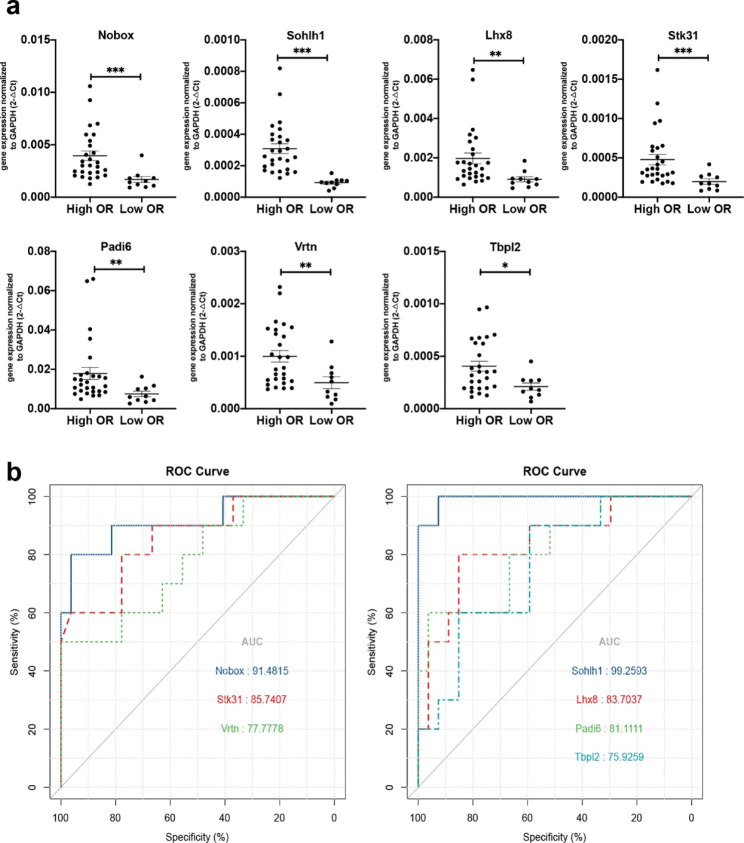
Relative expression and ROC analysis of candidate genes in OR comparison murine models. **(a)** mRNA relative expression of candidate genes in high and low OR group. * P < 0.05; ** P < 0.01; *** P < 0.001. **(b)** ROC curve of candidate genes to evaluate high OR levels

To investigate whether the expression of *Lhx8, Nobox, Sohlh1, Tbpl2, Stk31, Padi6*, and *Vrtn* possess the capacity to evaluate OR levels, qRT-PCR was performed to verify their expression in the contralateral ovary from each mouse. The relative expression level of all these genes was significantly higher in the high OR group than in the low OR group (Fig. 2a). ROC curve analysis was further performed to assess the capacity of each of these biomarkers to evaluate high or low OR. As shown in Fig. 2b; Table 1, the AUC for each gene was significantly greater than 0.5 (p < 0.05). The AUC for *Sohlh1* was 0.993 with a standard error of 0.010, and the sensitivity and specificity of the *Sohlh1* in evaluating OR capacity were 92.6% and 100%, respectively, at the chosen cut-off value of 0.00015527. These results suggested that all these genes have the capability to evaluate OR levels in mice.

### Correlation of mRNA expression levels with the primordial follicle pool

**Fig. 3:**
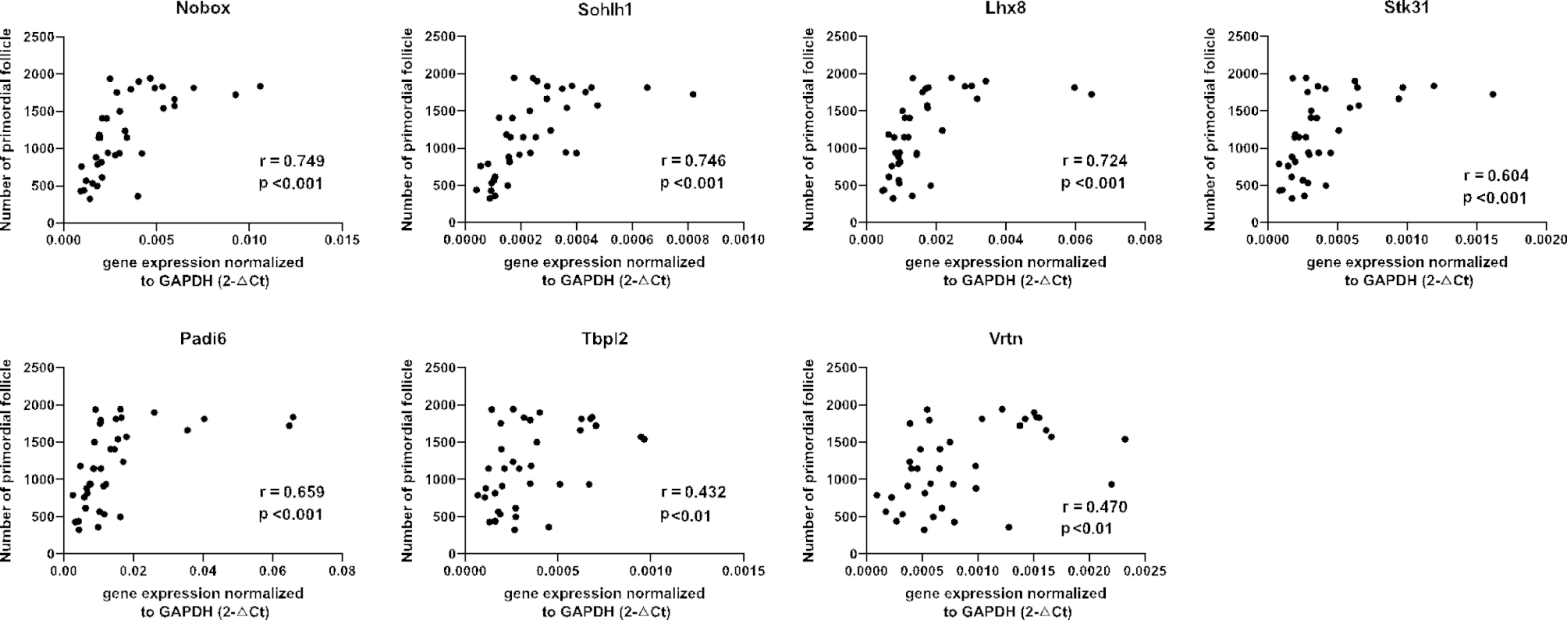
Relationship between the mRNA relative expressions of candidate biomarkers and primordial follicle numbers in murine paired ovaries. r, Pearson coefficient

Next, the correlation was further analyzed between the expression of candidate biomarkers and the number of the PFP counted in each mouse. All seven candidate biomarkers were significantly correlated with PFs (Fig. 3), yet their correlation coefficients varied. Among them, *Nobox*, *Sohlh1*, and *Lhx8* had a strong correlation (r > 0.7), whereas *Tbpl2* and *Vrtn* had a weaker correlation.

### Univariate linear analysis of candidate biomarkers to evaluate PFP

As shown in Fig. 3, the linear fit does not seem an optimal fit between gene expression form (2^−ΔCt^) and PFP. To investigate the optimal form of gene expression for linear regression analysis with PFP, the best-fit curves between the relative expression of candidate genes (2^−ΔCt^) and PFP were assessed via Curve Estimation using SPSS software. The results showed that the logarithmic model had the best goodness of fit between the relative expression of each gene and the number of PFs (Sup. Table 2). We depicted the expression levels of each gene in the form of -ΔCt to obtain more accurate fitting results in the subsequent linear regression analysis. Univariate analysis revealed that each of these potential biomarkers had a significant (p < 0.05) influence on PFP (Table 2). Among them, sohlh1 possessed the highest fitting coefficient (R^2^ = 0. 544) and Tbpl2 possessed the lowest (R^2^ = 0. 191).

### Stepwise regression analysis to identify the best model for evaluating the PFP

To identify the best model of these seven biomarkers for evaluating PFP, a multivariate linear stepwise regression analysis was performed with these seven genes (-ΔCt) as predictors and the number of the PFP as the dependent variable. The stepwise approach allowed us to identify the best combination of predictive variables by including all statistically significant independent variables according to their degree of contribution. Our analysis showed that the combination of *Sohlh1* and *Lhx8*, each acted as significant independent predictors (p < 0.05 and VIF < 5), exhibited the best fitting coefficient (R^2^ = 0. 597) for evaluating PFP among these seven biomarkers (Table 3, Sup. Table 3).

**Table 2:** Univariate linear analysis of candidate biomarkers to evaluate PFP

Gene (-ΔCt)	R^2^	B (95% CI)	Standard β	*P*
*Nobox*	0.537	439.160 (299.138 to 579.182)	0.733	**< 0.001**
*Lhx8*	0.506	419.064 (277.023 to 561.105)	0.711	**< 0.001**
*Sohlh1*	0.544	397.531 (272.555 to 522.506)	0.737	**< 0.001**
*Stk31*	0.386	329.701 (187.126 to 472.275)	0.622	**< 0.001**
*Tbpl2*	0.191	243.811 (71.479 to 416.143)	0.437	**0.007**
*Padi6*	0.420	326.886 (195.091 to 458.680)	0.648	**< 0.001**
*Vrtn*	0.229	241.578 (89.366 to 393.790)	0.478	**0.003**

R^2^, the coefficient of determination indicating the goodness-of-fit; B, regression coefficient; CI, Confidence Intervals; β, Beta coefficient; P values < 0.05 are in bold

**Table 3:** Multivariate linear stepwise regression to identify the best model of candidate biomarkers for evaluating PFP

Model	Gene (-ΔCt)	R^2^	R^2^ change	B	S.E. B	Standard β	*P*	VIF
1	*Sohlh1*	0.544	0.544	397.531	61.561	0.737	< 0.001	1
2	*Sohlh1*	0.597	0.544	250.982	90.407	0.466	0.009	2.375
	*Lhx8*		0.053	210.432	98.773	0.357	0.040	2.375

R^2^, the coefficient of determination; B, regression coefficient; S.E. B, standard error of regression coefficient; β, Beta coefficient; VIF, variance inflation factor

## Discussion

In the present study, we utilized an OR comparison model in mice to further investigate the validity of seven potential biomarkers identified by our previous bioinformatics analysis for assessing PFs, including *Sohlh1, Nobox, Lhx8, Tbpl2, Stk31, Padi6* and *Vrtn*. The results showed significant differences in the expression of the seven candidate genes in the OR comparison model. AUC analysis showed these seven biomarkers possessed the independent potential to assess OR levels. Further regression analysis between these biomarkers with PF showed that all seven genes had the independent potential to evaluate PFP, and the combination of *Sohlh1* and *Lhx8* had the best fit with PFs, suggesting that *Sohlh1* and *Lhx8* can be used as the optimal combinational biomarkers for rapid assessment of PFP in the mouse ovary.

The PFP is an essential subject in studies related to ovarian physiology and pathogenesis [4–6]. In clinical practice, the assessment of ovarian PFP in patients is mainly done indirectly by imaging and serology such as AMH, while there is no efficient method for the direct evaluation of ovarian tissue applied in clinical OTC, in vitro activation (IVA) and other ART [21–23]. In clinical OTC studies, ovarian tissues are processed to a specific size (e.g., 5 mm*5mm*1mm) and then frozen [21]. Several years later, before these ovarian tissues are rethawed with a series of treatments and re-transplanted back into the patient, the OR levels in the tissues are unknown to the physicians and patients, and the effectiveness of the transplantation is even less known. Generally, the assessment of the exact number of PFP in ovarian tissues based on microscopic counting is considerably subjective, and the number of follicles reported by different teams varies dramatically [7], which makes it conceivably challenging to apply for rapid assessment in clinical applications and animal studies. Therefore, a rapid and effective approach is urgently needed to evaluate OR levels in clinics. Our previous work has initially screened candidate genes in evaluating OR levels histologically [14], and this study further validated the feasibility of seven candidate genes to assess histological OR levels in combination with the classical histological assessment of PFP.

Quantitative real-time PCR (qRT-PCR) as a technology with high sensitivity and reproducibility has been applied in many areas, including clinical diagnostics, pharmacology, toxicology, and food safety [24–26]. In clinical diagnostics such as viral or bacterial infections, qRT-PCR techniques have been developed as diagnostic tools for rapid and accurate detection compared to traditional methods. In particular, the recent prevalence of the COVID-19 pathogen has reinforced the role of qRT-PCR as an irreplaceable tool for the early diagnosis of this disease, also highlighting the potential applicability of qRT-PCR that has been overlooked [27]. Therefore, in the present study, the qRT-PCR assay was used to verify further the validity of candidate biomarkers for PFP assessment based on our previous bioinformatics analysis. Our results confirmed that all seven genes could independently evaluate the number of PFP, and the combination of *Sohlh1* and *Lhx8* had an optimal fit with PFs, suggesting that *Sohlh1* and *Lhx8* could be used as the optimal biomarkers for rapid assessment of PFs in murine ovarian tissue.

Previous studies reveal that SOHLH1 and LHX8, as crucial oocyte-specific transcription factors, are essential in regulating postnatal folliculogenesis, especially in the earliest stages of primordial follicle activation [28–30]. Sohlh1, located on human chromosome 9 and mouse chromosome 2, encodes a basic helix–loop–helix transcription factor with homologs in humans and mice. Lhx8 is located on human chromosome 1 and mouse chromosome 3, and its encoded protein is a transcription factor containing a cysteine-histidine LIM structural domain involved in the morphogenesis of several organs [31]. In the murine ovary, both *Sohlh1* and *Lhx8* are expressed specifically in both embryonic and postnatal female germ cells starting at embryonic day 13.5 (E13.5). Despite a slight difference in the expressions of *Sohlh1* and *Lhx8* in oocytes at different stages of the postnatal follicle, both were relatively highly expressed at early stages (primordial and primary stages)[32]. Conditional deficiency of either *Sohlh1* or *Lhx8* in the oocytes of primordial follicles causes massive primordial oocyte activation and postnatal oocyte depletion leading to infertility in mice [28].

As for the regulatory relationship between *Sohlh1* and *Lhx8*, ChIP experiments of newborn mouse ovaries conducted by Pangas et al. in earlier years suggested that *Lhx8* is likely one of the direct downstream target genes of SOHLH1 through the E box elements in their promoters [32]. While a recent study conducted by Wang et al. shows that SOHLH1 and LHX8 can cross-regulate each other and physically bind to each other with Figla to form a nuclear complex in oocytes to regulate transcriptional activity during early oocyte growth and differentiation [28].

The above studies on the molecular mechanisms of *Sohlh1* and *Lhx8* further demonstrated the reliability and feasibility of *Sohlh1* and *Lhx8* could use as prominent biomarkers to estimate PFP in our present findings. However, the explicit mechanism of maintaining quiescent follicles by these transcription factors still needs further study. We believe it is feasible for the validity of such assessment tools in models like the chemically induced premature ovarian failure (POF) model or OTC model, but of course, the exact validity still needs to be verified in each specific model with a considerable number of mice. Besides, although the genes in this study are highly homologous to humans, the feasibility of the present findings in human ovarian tissue slices requires additional validation with large sample sizes.

In conclusion, the results of this study suggest that *Sohlh1*, *Nobox*, *Lhx8*, *Tbpl2*, *Stk31*, *Padi6* and *Vrtn* these seven biomarkers possess independent potential to evaluate PF numbers, and the combination of *Sohlh1* and *Lhx8* can be used as the optimal biomarkers for rapid assessment of PFs in the mouse ovary. Our findings provide a new perspective for evaluating PFP of ovaries in animal studies and clinics.

## Electronic supplementary material

Below is the link to the electronic supplementary material.

Supplementary Material 1

## Data Availability

The data that support the findings of this study are available from the corresponding author upon reasonable request.
