# Supplementary material for: Sohlh1 and Lhx8 are prominent biomarkers to estimate the primordial follicle pool in mice
Source: Reprod Biol Endocrinol. 2023 May 16;21:46. doi: 10.1186/s12958-023-01097-3 (PMC10186293; doi:10.1186/s12958-023-01097-3)
Supplement: Supplementary file 1 — Supplementary Material 1 [file 12958_2023_1097_MOESM1_ESM.docx]

Supplementary Material

# Supplementary Table1. Primers used for qPCR validation

| Gene (mouse) | Forward Sequence | Reverse Sequence |
| --- | --- | --- |
| *Lhx8* | AGCACAGTTCGCTCAGGACAAC | GCTGAGGAAGAATGGTTGGGAC |
| *Nobox* | CGTTCCTGGCAGTGACAGCATA | GGAATGAACCCAACTGGCTGCT |
| *Sohlh1* | GCCAAACCATCTGCTGTGTCTC | AAGGTCTCTCCAGCAGCTCTGA |
| *Tbpl2* | ACTCCAATGCCTTACCTGTGGC | GCCAGATTTGCAGTGGAAACTAC |
| *Padi6* | CTGAGCGAGAAGAGCAAAGTGC | ATGACACCGTCTTGTGAGGAGC |
| *Stk31* | CGTGTGTAGGAACCAGGCTGAA | GGACCCTTCATCCAACACTTGC |
| *Vrtn* | ACCAAGAGCACCTTCTACCGCT | GAACTGCTGCAATGGCACAAAGC |

# Supplementary Table2. Curve estimation between the relative expression of candidate biomarkers (2^-ΔCt) and the PFP

| Gene | Linear | | Logarithmic | | Exponential | | Logistic | |
| --- | --- | --- | --- | --- | --- | --- | --- | --- |
|  | R^2^ | *P* | R^2^ | *P* | R^2^ | *P* | R^2^ | *P* |
| *Nobox* | 0.424 | <0.001 | **0.537** | <0.001 | 0.357 | <0.001 | 0.357 | <0.001 |
| *Lhx8* | 0.350 | <0.001 | **0.506** | <0.001 | 0.280 | <0.01 | 0.280 | <0.01 |
| *Sohlh1* | 0.416 | <0.001 | **0.544** | <0.001 | 0.400 | <0.001 | 0.400 | <0.001 |
| *Stk31* | 0.298 | <0.001 | **0.386** | <0.001 | 0.258 | <0.01 | 0.258 | <0.01 |
| *Tbpl2* | 0.183 | <0.01 | **0.191** | <0.01 | 0.159 | <0.05 | 0.159 | <0.05 |
| *Padi6* | 0.276 | <0.01 | **0.420** | <0.001 | 0.225 | <0.01 | 0.225 | <0.01 |
| *Vrtn* | 0.199 | <0.01 | **0.229** | <0.01 | 0.163 | <0.05 | 0.163 | <0.05 |

# 3 Supplementary Table3. Other variables excluded in multiple linear stepwise regression

| Model | | Beta | t | Sig. | Partial Correlations | Covariance statistics | |
| --- | --- | --- | --- | --- | --- | --- | --- |
|  |  |  |  |  |  | tolerance | VIF |
| 1 | *Nobox* | 0.388^b^ | 1.970 | 0.057 | 0.320 | 0.311 | 3.213 |
|  | *Lhx8* | 0.357^b^ | 2.130 | **0.040** | 0.343 | 0.421 | 2.375 |
|  | *Stk31* | 0.085^b^ | 0.442 | 0.661 | 0.076 | 0.357 | 2.798 |
|  | *Tpbl2* | -0.140^b^ | -0.885 | 0.383 | -0.150 | 0.522 | 1.917 |
|  | *Padi6* | 0.255^b^ | 1.616 | 0.115 | 0.267 | 0.502 | 1.992 |
|  | *Vrtn* | 0.056^b^ | 0.390 | 0.699 | 0.067 | 0.640 | 1.562 |
| 2 | *Nobox* | 0.215^c^ | 0.877 | 0.387 | 0.151 | 0.198 | 5.051 |
|  | *Stk31* | -0.262^c^ | -1.123 | 0.270 | -0.192 | 0.216 | 4.626 |
|  | *Tpbl2* | -0.178^c^ | -1.180 | 0.246 | -0.201 | 0.515 | 1.940 |
|  | *Padi6* | -0.008^c^ | -0.030 | 0.976 | -0.005 | 0.191 | 5.236 |
|  | *Vrtn* | 0.027^c^ | 0.195 | 0.846 | 0.034 | 0.634 | 1.578 |

b. Predictor variables in the model: *Sohlh1*
c. Predictor variables in the model: *Sohlh1, Lhx8*
